# Deep learning applications in protein crystallography

**DOI:** 10.1107/S2053273323009300

**Published:** 2024-01-01

**Authors:** Senik Matinyan, Pavel Filipcik, Jan Pieter Abrahams

**Affiliations:** a Biozentrum, Basel University, Basel, Switzerland; b Paul Scherrer Institute, Villigen, Switzerland; Institute of Crystallography - CNR, Bari, Italy

**Keywords:** protein crystallography, deep learning, artificial intelligence, machine learning

## Abstract

Deep learning techniques can recognize complex patterns in noisy, multidimensional data. In recent years, researchers have started to explore the potential of deep learning in the field of structural biology, including protein crystallography. This field has some significant challenges, in particular producing high-quality and well ordered protein crystals. Additionally, collecting diffraction data with high completeness and quality, and determining and refining protein structures can be problematic. Protein crystallographic data are often high-dimensional, noisy and incomplete. Deep learning algorithms can extract relevant features from these data and learn to recognize patterns, which can improve the success rate of crystallization and the quality of crystal structures. This paper reviews progress in this field.

## Abstract 2

Deep learning applications are increasingly dominating many areas of science. This paper reviews their relevance for and impact on protein crystallography.

## 1. Introduction

Protein crystallography is a crucial tool for understanding the three-dimensional structures of proteins (Bücker *et al.*, 2020 ▸). The vast majority of the protein structures deposited in the Protein Data Bank (Berman *et al.*, 2000 ▸) were solved with crystallographic methods (∼85% of the deposited structures and around 10 000 structures annually). Exciting advancements in the field, such as X-ray free-electron lasers (XFELs) (Chapman *et al.*, 2011 ▸; Tenboer *et al.*, 2014 ▸) and MicroED (microcrystal electron diffraction) (Nederlof *et al.*, 2013 ▸), have significantly improved the efficiency of determining protein structures, even for sub-micron-sized crystals.

However, several key challenges persist in protein crystallography, the main one being the production of high-quality and well ordered protein crystals. Additionally, extracting accurate protein structures from diffraction data remains a complex task. Fortunately, deep learning techniques have emerged as a promising solution to address these limitations.

Deep learning is a branch of machine learning (ML) that employs artificial neural networks to learn complex patterns from data (Sarker, 2021 ▸). Analogous to biological neural networks, these consist of multiple layers of interconnected nodes, each layer representing a different level of abstraction. This allows the development of algorithms and network architectures that can be readily applied to various types of data in order to create and/or optimize a model – an ML program tailored for the task. ML models are created using training data that can be labeled or unlabeled. One of the major risks in preparing an ML model is that training data may not be sufficiently representative of the problem at hand, resulting in a neural network that is biased by preconceptions. Since the strength of deep learning lies in its ability to analyze complex and high-dimensional data, it has significant relevance for the analysis of protein crystallography data.

In this review, we explore the application of deep learning techniques to overcome the challenges in protein crystallography. By harnessing the power of deep learning, the field of protein crystallography is unlocking new possibilities, leading to a deeper understanding of protein structures and their functional implications. But first, we briefly summarize the history of deep learning, provide some essential definitions and describe those traditional ML and deep learning methods that have found applications in protein crystallography.

### 1.1. Deep learning as a branch of machine learning

The roots of ML can be traced back to the early 1950s, when researchers started exploring the concept of intelligent machines. Inspired by the human brain’s neural networks, researchers began developing artificial neural networks in the 1980s. However, progress was initially hindered by limited computational resources and a scarcity of large-scale data, despite the introduction of the efficient backpropagation algorithm for training multi-layer neural networks (Karhunen *et al.*, 2015 ▸).

In the early 1990s, various ML algorithms, such as support vector machines (SVM), decision trees and ensemble methods, gained momentum. These data-driven approaches started to outperform rule-based systems. While neural networks with multiple hidden layers have been explored for decades, a pivotal work by Hinton *et al.* (2006 ▸) introduced effective techniques for training these networks (Hinton *et al.*, 2006 ▸). The introduction of deep belief networks marked the beginning of the deep learning era.

As the power of computational resources increased, and large-scale data sets became more readily available, deep learning increased in popularity. Subsequent advancements in the field focused on optimizing the architecture of neural networks in terms of depth, connectivity and node properties, recognizing their crucial role in efficiently solving specific tasks.

Today, deep learning has become the main approach in the broad area of ML. It seeks to mimic human reasoning and apply that knowledge in a broader context (Sarker *et al.*, 2021 ▸). By uncovering intricate patterns and models within highly complex systems that defy traditional analysis, deep learning has the potential to revolutionize scientific research.

### 1.2. Some definitions

ML techniques can be categorized into three main types: supervised learning, unsupervised learning and reinforcement learning.

In supervised learning the algorithm is trained on a data set of input–output pairs that are labeled by a human domain expert. The algorithm learns to map the input data to the ground truth by minimizing a loss function (for details, see Appendix *C*2). The goal of supervised learning is to accurately predict the target variable for new, unseen inputs.

In unsupervised learning, the algorithm is trained on an unlabeled data set and seeks to uncover hidden patterns or relationships within the data without the guidance of specific labels. By exploring the inherent structure of the data, unsupervised learning algorithms reveal valuable insights.

In reinforcement learning the algorithm learns to make decisions in dynamic environments. These algorithms learn to maximize cumulative rewards over time, based on the feedback they receive from the environment. By exploring different actions and their consequences, reinforcement learning agents discover optimal strategies for achieving desired outcomes.

The two primary ML tasks are classification and regression. The goal of classification is to assign discrete labels or categories to input data points. Regression aims to predict continuous target values.

Classification models are typically evaluated using metrics such as accuracy, precision, recall, F1-score, area under the ROC (receiver operating characteristic) curve (AUC-ROC) and Matthew’s correlation coefficient (MCC) [for details, see Appendix *B*1, equations (1)–(5)]. (The MCC has no relationship to Matthew’s coefficient *V*_M_ in protein crystallography, which is the crystal volume per unit of protein molecular weight.) These metrics provide insights into the model’s performance in terms of correctly classifying different instances.

Regression models are evaluated using metrics such as mean absolute error (MAE), mean-squared error (MSE), root-mean-squared error (RMSE) and *R*-squared [for details, see Appendix *B*2, equations (6)–(8)]. These metrics measure the deviation between the predicted continuous values and the actual target values, providing an assessment of the model’s predictive accuracy.

### 1.3. Data assessment

An efficient data assessment workflow is vital for proper model development and for evaluating the robustness of the model. The source of the data and the nature of the variables influence the selection of preprocessing steps, and the selection of the most effective ML algorithm. Investigating the variable distributions can reveal the intricate relationships between them and potential multi-collinearity issues, which significantly impact model performance, necessitating transformations or normalization for certain algorithms. Feature extraction and recognition of potential outliers often require domain expertise, because automatic feature extraction may compromise the interpretability of the model. A significant body of exploratory data analysis and initial assessment techniques is available in a previous review on this topic (Vollmar & Evans, 2021 ▸).

### 1.4. Traditional ML and deep learning

**Figure 1:**
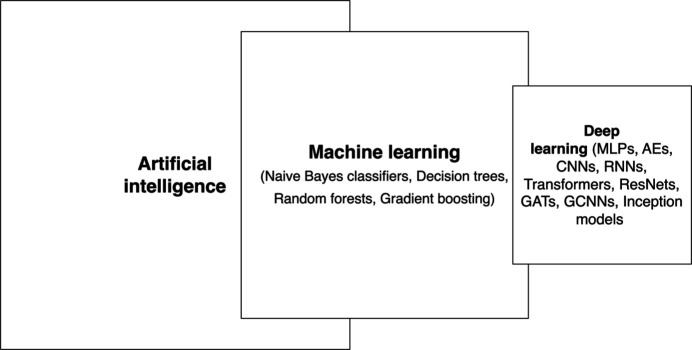
Traditional and deep learning architectures used in different steps of protein crystallography. The exact details of the architectures and working principles are summarized in Appendices *A* [App appa] and *B* [App appb].

Traditional ML algorithms offer a variety of solutions, but with inherent limitations, and deep learning was developed to address some of these challenges. Nevertheless, traditional ML has been applied extensively and effectively in many applications. Some of these have also been applied to the field of protein crystallography, and they, together with deep learning models currently used in this field, are summarized in Fig. 1 ▸.

We review the application of deep learning architectures in key steps of protein crystallography. In every section, we mention traditional ML algorithms to contrast them with the advancements introduced by deep learning.

## 2. Deep learning and key steps of protein crystallography

### 2.1. Protein crystallization propensity

One of the key advantages of deep learning techniques in protein crystallography is their potential to predict the targets that are more likely to crystallize (Mizianty & Kurgan, 2011 ▸). Protein crystal quality is an important factor in the success of structure determination, as poor-quality and disordered crystals can lead to inaccurate results. Only a minority of target proteins (4.6%) produce crystals of sufficient quality, and failure to crystallize remains the prime bottleneck in protein crystallography (Mizianty & Kurgan, 2011 ▸). First, we summarize current traditional ML approaches, and then we discuss how their limitations have been addressed by deep learning.

Several *in silico* traditional ML and analytical approaches have been developed to address the crystallizability, including but not limited to *CRYSTALP2* (Kurgan *et al.*, 2009 ▸), *PPCpred* (Mizianty & Kurgan, 2011 ▸), *XtalPred-RF* (Jahandideh *et al.*, 2014 ▸), *TargetCrys* (Hu *et al.*, 2016 ▸), *Crysalis* (Wang *et al.*, 2016 ▸), *fDETECT* (Meng *et al.*, 2017 ▸), *BCrystal* (Elbasir *et al.*, 2020 ▸) and *DCFCrystal* (Zhu *et al.*, 2021 ▸) (see details of traditional ML algorithms in Appendix *A*1). The first attempts *CRYSTALP2* and *PPCpred* (predictor of protein production, purification and crystallization) predict the tendency to produce diffraction-quality crystals based on a wide range of input features: energy and hydro­phobicity indices, amino acid composition and sequence, isoelectric point, predicted disorder, secondary structure and solvent accessibility, and content of certain buried and exposed residues. *XtalPred-RF* consists of a series of independent random forest (RF) classifiers, and performs extensive feature extraction to better approximate the initial information domain. *TargetCrys* utilizes a two-layer SVM, where the first-layer decisions, based on the respective feature sets, are further ensembled by a second layer of SVM. The *Crysalis* integrated webserver not only predicts the crystallization propensity, but also helps the developers to design point mutations for better crystallization outcome. Individual step prediction is also possible in the webserver *fDETECT*, which uses a logistic regression method and is a fast protein production, purification and crystallization predictor. The *BCrystal* webserver takes advantage of the ‘gradient boosting machine’ (XGBoost) to estimate protein crystallization propensities. It performs feature pruning automatically, reducing the risk of overfitting and explains the predicted class label for each protein based on its corresponding feature using the SHapley Additive exPlanations (SHAP) algorithm (Lundberg & Lee, 2017 ▸). *DCFCrystal* is based on a cascaded RF and predicts individual steps of the crystallization process: production of protein material, purification and production of crystals. The single-stage variant of *DCFCrystal* (*MDCFCrystal*) is specifically designed for membrane proteins.

The performance of all these traditional ML methods is highly determined by the extent of feature extraction, requires domain expertise, and fluctuates over a wide range. Most existing predictors tend to sequentially merge diverse features. While there is evidence that combining features from multiple sources can sometimes enhance prediction accuracy, it does not always guarantee better results and may introduce redundant information in the feature space. This redundancy can potentially weaken the predictor’s accuracy for new data sets. These limitations can be addressed by leveraging the potential of deep learning architectures.

*DeepSol* has a deep learning architecture based on a convolutional neural network (CNN, for details of this architecture, see Appendix *A*2.3), which was trained to predict protein solubility from the input protein sequence and additional features. These features include sequence length, molecular weight, the aliphatic indices, the average hydro­phobicity value (GRAVY, grand average of hydro­pathy), the fraction of turn-forming residues, and structural features predicted from the protein sequence using SCRATCH (Magnan & Baldi, 2014 ▸). The proposed solution could reach a MCC of 0.55. *DeepSol* was highly selective in identifying insoluble proteins, outperforming the previous state-of-the-art solutions (Khurana *et al.*, 2018 ▸).

The *DeepCrystal* framework (Elbasir *et al.*, 2019 ▸) also identifies patterns indicative of successful protein crystallization by leveraging the discriminative power of CNNs for analyzing amino acid sequences. The protein sequence is given as a one-hot encoded vector with a length of 22 (20 for amino acids, 1 for gap and 1 for ambiguous amino acids), with only 1 bit active for the *i*th amino acid in a protein sequence. The data are passed through a filter that removes redundant sequences and the crystallization prediction is defined as a binary classification problem. The framework takes advantage of the high degree of correlation between the primary sequence of a protein and its propensity to crystallize by training a multi-scale CNN on a data set of proteins with known crystallization outcomes. The accuracy of the *DeepCrystal* model was 0.83 and the MCC value was 0.66 on the evaluated data set. *DeepCrystal* is reported to achieve an accuracy that was 5–30% higher than traditional sequence-based predictors and is better at predicting crystallizability of shorter proteins. *DeepCrystal* was made publicly available through a webserver and accepts protein sequences in FASTA format (https://github.com/elbasir/DeepCrystal).

Another deep learning framework, *CLPred* (Xuan *et al.*, 2020 ▸), takes a raw protein sequence as input and converts the amino acids into a ‘*k*-mer’ vector representation through a word embedding layer. A ‘*k*-mer’ is a subsequence of consecutive amino acids within a protein sequence. For example, for a sequence ‘ABCD’ and a *k*-mer size of 3 (*k* = 3), there are two 3-mers (‘ABC’ and ‘BCD’). Next, the high-frequency *k*-mer features are identified through a CNN layer. Finally, the *k*-mer features are fed into a bidirectional recurrent neural network with long short-term memory LSTM (BLSTM) (for details, see Appendix *A*2.4), which captures the long-range interaction information between *k*-mer amino acids and generates predictions. *CLPred* outperformed other evaluated predictors such as *fDETECT*, *TargetCrys*, *PPCpred*, *CRYSTALP2* and *DeepCrystal*, showing the highest MCC of 0.700 and the highest accuracy of 0.851. When the authors combined *CLPred* last-layer embeddings with 641 additional features, including 8-state secondary structure (SS), fraction of exposed residues (FER), disorder and hydro­phobicity, the performance of the network was even better. The paper shows that deep convolutional or fully connected layers are capable of extracting more complex features. However, further additions of computing layers may downgrade the prediction performance. Furthermore, LSTMs are best at modeling temporal sequences, and their applicability may be limited in protein sequences with complex three-dimensional structures. LSTMs cannot execute calculations in parallel and have a lower time-efficiency compared with *DeepCrystal*.

*ATTCry* is an attention-based neural network for crystallization propensity prediction. *ATTCry* can extract both local and global features of protein sequences. For this purpose, it uses multi-head self-attention layers (for details, see Appendices *A*2.6 and *A*2.7) and integrates that information with a multi-scale CNN, to obtain a more complex global spatial dependence of protein structure (Jin *et al.*, 2021 ▸). Each head captures the long-distance dependence and the CNN extracts local *k*-mer structures. The proposed framework achieved a prediction accuracy of 0.866 and an MCC score of 0.716. Ablation studies showed that both multi-scale CNN layers and multi-head self-attention layers appeared to be essential for crystallization prediction. In all the aforementioned cases, the length of the protein sequence was limited to 800 amino acids.

Another approach, *SADeepcry*, uses a latent representation of 9139-dimensional physico-chemical, sequence-derived and ‘disorder’ features and an optimized multi-head self-attention mechanism to extract a complex representation of protein structural and chemical features. (The term ‘latent space’ refers to hidden or underlying features or variables in data which are not directly observable, but can be inferred or learned through ML models.) These features are encoded using an autoencoder (AE) (for details, see Appendix *A*2.2). The predictor is a multi-layer perceptron (MLP) (for details, see Appendix *A*2.1) module, where the authors concatenate the output by the self-attention module and latent space from the AE (Wang & Zhao, 2022 ▸). The model showed comparable results with respect to *ATTCry* and *DeepCrystal*, and achieved the best results in accuracy and AUC-ROC metrics. It achieved an MCC of 0.748 and an accuracy of 0.877.

The mentioned techniques build the interaction knowledge of residues at sequence level rather than structure level. However, extracting the structure-level information may be a better predictor for protein crystallization. For this, a graph attention network (for details, see Appendix *A*2.9) was developed (*GCmapCrys*), that includes residue-interaction knowledge in crystallization behavior (Wang *et al.*, 2023 ▸). Sequence-based features are the nodes of the protein graph, and the predicted contact map of the protein serves as the edge between the nodes. The sequence-based features include a position-specific scoring matrix, the physico-chemical and biological properties from the AAindex database (Kawashima & Kanehisa, 2000 ▸), the average hydro­phobicity value (GRAVY), and other characteristics. After three consecutive graph attention layers, the global pooled representation is given to two consecutive fully connected layers, where the second layer uses a sigmoid activation layer (for details of various activation functions, see Appendix *C*1) to predict crystallization propensity. *GCmapCrys* was benchmarked using different data sets, and its accuracy was at least 0.71 with an MCC value higher than 0.33. Further analysis showed predicted structure-based coding (PSBC) to be the most important complementary feature for crystallization propensity prediction.

Overall, while traditional techniques for predicting protein crystallization continue to play an essential role, deep learning models have introduced a revolutionary approach to the field. These models capture intricate patterns and relationships between protein sequences and crystallization success, overcome the hand-crafted feature representations and can help researchers identify the most promising candidates for crystallization experiments. This predictive capability can accelerate the process of protein structure determination and reduce the experimental cost. Nevertheless, up until now, deep learning models complement, rather than replace, traditional methods. We consider it essential, for further progress, to establish means of independent validation of the various deep learning approaches, in view of differences in benchmarked data sets and inherent interpretability constraints of these models.

### 2.2. Crystallization monitoring

Protein crystallization is a complex process that requires the determination and optimization of a wide range of parameters (Liu *et al.*, 2008 ▸). With ultra high throughput methods becoming increasingly prevalent, there is a growing need to automatically assess massive volumes of image data from crystallization trials. The problem is exacerbated by the fact that crystallographers often disagree on the class of images: when 16 crystallographers assessed 1200 trial images, they reached an overall agreement of only 70% (Wilson, 2006 ▸). Moreover, assigning different scores to the same image on different occasions is also common. To address these challenges, there is an urgent need for systems that can improve existing image-analysis pipelines by automatically and accurately throwing out the vast majority of crystal-negative trials while minimizing the risk of rejecting crystal-positive trials. In an ideal scenario, the system would achieve a perfect score of zero false negatives, as missing a valuable crystallization condition could potentially jeopardize the entire structure determination project. Additionally, it should maintain a low ratio of false-positive results within acceptable limits irrespective of the imaging platform used, mimic the skills of an experienced crystallographer for recognizing different types of crystals, ignore technical failures, and learn from experience. In this context, the application of deep learning to monitor crystal growth is limited by the requirement of large amounts of manually labeled data, which can contain annotation errors and biases, and are considered error-prone due to discrepancies in human labeling (Bolya *et al.*, 2020 ▸). We first summarize traditional ML approaches for crystallization monitoring.

Researchers analyzed 319 112 crystallization trial images from 150 solved structures deposited at the Protein Data Bank, using the boosting technique to find well diffracting crystals (Liu *et al.*, 2008 ▸). The approach finds lines and textures indicative of crystals and nanocrystals and could reach an AUC-ROC score of 0.92. Each square of the image encodes a feature vector of 466 values and is propagated differently through the alternating decision tree. The maximum score over all squares is taken as the image score which is then used for discrimination. Feature extraction relies on Gabor wavelet responses to detect edges and texture (Pan *et al.*, 2006 ▸). Selecting the top 20% ranked images of each set was successful in picking an image that led to a good crystal in 145 out of 150 cases, proving the effectiveness of the selection method.

Traditional ML approaches leveraged the potential of edge detection followed by curve tracking (Bern *et al.*, 2004 ▸), employing a two-tier cascade classifier using naïve Bayes and RF (Hung *et al.*, 2014 ▸; Cumbaa & Jurisica, 2010 ▸). The authors used massive feature engineering and a set of 165 351 hand-scored images to train an RF classifier for crystallization detection. In both 10-way and 3-way classifiers, ‘precipitates’ and ‘clear drops’ were easily recognized. 80% of crystals were correctly detected in the classification task as well. However, the classifier rejected all observations within the ‘phase & precip’ category. An extensive list of other analytical and traditional ML techniques for crystallization monitoring is described elsewhere (Sigdel *et al.*, 2013 ▸).

While traditional ML methodologies attempt to alleviate the crystallization monitoring challenge, the degree of precision obtained is not sufficient to replace manual inspection. Observed external regularity, as captured in images by strong edges, symmetry and polygonal shapes, may not always correlate with high-resolution diffraction. Employing advanced deep learning techniques, described here, could remove this shortcoming.

*CrystalNet* employs a CNN that was trained on 163 894 high-resolution grayscale labeled images of protein crystallization trials on 1536-well plates, and achieved a 0.908 in accuracy, with an AUC-ROC of 0.9903 for crystal class classification (Yann & Tang, 2016 ▸). *CrystalNet*’s first-layer filters catch useful edge information, giving a clue as to the features most discriminative for the classification. As expected, sharp images were required, as low-resolution, blurred image data resulted in a significant drop in accuracy. When operating in real time, *CrystalNet* could handle more than 750 images per second, making it a good choice for the automated evaluation of microarray plates.

*CrystalNet* and other, more sophisticated CNN architectures, using the topology of AlexNet (Krizhevsky *et al.*, 2012 ▸), VGG (Simonyan & Zisserman, 2014 ▸) Inception-V3 (Szegedy *et al.*, 2016 ▸) and ResNet (He *et al.*, 2016 ▸) (for details, see Appendices *A*2.10 and *A*2.11), were benchmarked for crystallization monitoring (Ghafurian *et al.*, 2018 ▸). A training data set of 486 000 images was manually annotated into ten classes. The data set was augmented^1^ to mitigate class imbalance (class imbalance refers to a situation in data sets where one class significantly outnumbers the others, potentially leading to biased model predictions). The highest-performing CNNs were ResNet-56, ResNet-32 and Inception-V3, with testing accuracy of 0.814, 0.806 and 0.794, respectively. Due to the use of residual blocks, ResNet-56, even though it is the deepest network, did not suffer from depth-related degradation (for details of vanishing and exploding gradients, see Appendix *C*6) and showed the best results. Unlike the ResNet architecture, the addition of more layers to VGG resulted in a small decrease in accuracy. ‘Micro crystal’ and ‘medium crystal’ classifications showed the most significant variability across architectures: ResNet-56 showed an accuracy of 0.611, while *CrystalNet* only managed 0.493. ‘Small crystal’ appears to be the hardest class to identify for most architectures, with the lowest average accuracy.

The Inception-V3 architecture was also used in another study, trained on the Machine Recognition of Crystallization Outcomes (MARCO) initiative data set (Bruno *et al.*, 2018 ▸). The MARCO collaboration of five institutes collects and shares a large data set of images of crystallization trials for improving analysis techniques. The MARCO data set contains images produced by imagers of two different manufacturers and from in-house-developed systems, all with different optics. The four categories in this study were: ‘clear’, ‘precipitate’, ‘crystal’ and ‘other’. The data were augmented prior to training. During the relabeling phase, crystallographers were asked to relabel the images that disagreed strongly with the classifier. The MARCO model reached an accuracy of 0.942 with a mere 0.3% improvement after the relabeling. The study demonstrates that the model can effectively classify images of crystallization trials, independent of the systems used to create them. This approach offers consistency and efficiency, making it suitable for high-throughput settings and data mining of past image repositories. Although high classification rates were reported for the MARCO model, using images from multiple platforms, transferability remains an issue. In cases where the model gives inaccurate results, retraining the model on local data improved classification accuracy even if the training was performed on a significantly smaller data set (Milne *et al.*, 2023 ▸). Future efforts should focus on increasing the robustness and versatility by maintaining consistent performance across various experimental conditions, sharing the weights of the model and allowing transfer learning.

To facilitate protein crystallization experiments, an automated system called Real Time Protein Crystal Monitoring System (RT-PCMS) was developed (Sengar *et al.*, 2022 ▸). It has precise robotics and a custom-designed motorized microscopic imaging system capable of capturing multi-focus composite images of protein crystals in droplets across multiple wells. It is a high-throughput system for imaging crystals in 24- or 96-well crystallization plate formats, and monitoring their growth at frequent time intervals. In multi-well plates, where images may be captured under different illumination conditions, crystals appear at varying depths of field, and crystal growth phases may vary. To address these challenges, a multi-directional contourlet-based segmentation algorithm was implemented for extracting crystal features. To increase the overall depth of field of the scanned well, a fusion algorithm was used combining non-subsampled wavelet transform (NSWT) and a guided filter. The last step was the adoption of the Inception-V3 network. Compared with the MARCO model, the modified network has fewer coefficients, potentially allowing faster classification.

Microfluidic technology has emerged as a promising tool for screening crystallization conditions (Huang *et al.*, 2022 ▸). It offers several advantages including high speed, low reagent consumption and low cost. However, several bottlenecks exist, including insufficient high-speed data analysis, lack of time-resolved information, and inadequacy in yielding large enough crystals for direct use. Moreover, nanolitre-scaled trial volumes may have a reduced nucleation rate (Bodenstaff *et al.*, 2002 ▸), and can potentially augment the probability of ‘false negatives’ or ‘false positives’ (Maeki *et al.*, 2014 ▸).

The Deep Learning-Aided High-Throughput Programmable Microlitre-Droplet System (DL-HTPMS) was designed to address some of these limitations by combining microfluidics and deep learning. This system enables efficient screening of protein crystallization conditions in microlitre-scaled droplets, while also providing time-resolved information on protein crystalline states. To accelerate the screening process, a dense convolutional neural network (DenseNet) model was constructed to classify the different crystalline states or morphologies. This model exhibited high accuracy and recall (0.993). The temporal diagrams demonstrated high consistency (∼93%) with those obtained in the scale-up experiment.

Deep learning has been used for assessing and improving a novel approach that uses bioconjugate-functionalized nanoparticles (McCue *et al.*, 2023 ▸). The goal was to assess the effect of such nanoparticles on the nucleation rate and induction time of protein crystallization, particularly at low protein concentration conditions. A custom CNN-enabled emulsion crystallization setup for accurately quantifying nucleation parameters allowed the capture of a wealth of data regarding the crystallization process and prediction of the outcomes under different conditions. While nucleation of protein crystals has been studied for decades, significant breakthroughs in terms of robust, predictable and general protocols for improving crystal growth by including crystal nucleants, were scarce. Presumably, the high number and unpredictable nature of potentially relevant parameters have been a limiting factor, and it is therefore possible that deep learning may be able to extract complicated, hitherto overlooked, patterns and correlations.

Deep learning has a role in advancing protein crystallization monitoring, enabling efficient, accurate and quick identification of crystal formation. One of the main problems is that imagers may not be able to capture crystal formation at sufficiently high resolution and contrast, because of the experimental specifics of crystal screening. Images of more or less spherical droplets may suffer from distortions and uneven light transmission, and crystals may grow anywhere within a droplet, so a through-focus series may be required for observing the early stages of crystal growth. These shortcomings are the most likely explanation for the discrepancies found in expert human assessments of images of crystallization trials. Additional image modalities and more accurate and detailed three-dimensional imaging are likely to be required for further improvements. With these provisos, we consider that current deep learning approaches can be highly effective in both high-throughput settings and for data mining of past image repositories.

### 2.3. Diffraction data collection

Deep learning techniques have also been used for automating diffraction data processing and analysis in protein crystallography. The introduction of XFELs as a source of extremely bright and short pulses of X-ray radiation allowed the study of proteins that are difficult to crystallize or that degrade quickly under the intense X-ray radiation used in traditional crystallography methods. The femtosecond XFEL sources allow a ‘diffract before destroy’ approach, and radiation damage effects are significantly evaded (Bücker *et al.*, 2020 ▸). In this scenario, it is important to have data processing capabilities that give real-time feedback so that the characteristics of the experimental results can be tracked. By analyzing data quickly, key indicators such as the rate of increase in reciprocal-space coverage can be monitored, allowing experimental parameters to be adjusted before the sample and beam time are exhausted. However, providing sufficient computing resources for real-time analysis is a challenge. As data collection capacity increases, it is important to ensure that the data production rates do not exceed the speed of analysis. To prevent overloading the network and data processing resources with useless data, a screening tool that can quickly identify and store patterns with Bragg spots is vital (Ke *et al.*, 2018 ▸).

An AlexNet type of deep learning architecture was used for classifying diffraction frames as ‘hit’, ‘maybe’ or ‘miss’, with ‘maybe’ indicating a small number of Bragg spots (Ke *et al.*, 2018 ▸). For training, 2000 center-cropped images were preprocessed by local contrast normalization and were augmented. The data sets were chosen from a diverse range of imaging detectors, beam energies and sample delivery methods to ensure a representative cross section. The training data set included crystals with different space groups and unit-cell parameters. Two methods provided the reference for the training: annotation by a human expert, and an automated spotfinder in conjunction with thresholding. The accuracy of CNN classification was largely influenced by the quality of the annotated data used to train the network. With the exception of one data set, the confidence level of most correctly classified images was above 90%. When the training data set had a limited number of images with clearly visible and/or a small number of identifiable Bragg spots within the images, the CNN tended to have lower accuracy. Additionally, the accuracy was affected by factors such as the type of detector used, beam properties and sample preparation methods. Preprocessing the data using local contrast normalization was crucial for accurate CNN predictions. Without contrast adjustment, the CNN training was negatively affected by artifacts and background noise. The most informative pixels for the CNN classification not only covered the Bragg spots but also included their surrounding area, assisting the CNN in recognizing the presence of Bragg diffraction against the background.

*DeepFreak* was developed for selecting diffraction patterns for downstream analysis (Souza *et al.*, 2019 ▸). Three types of classifiers were trained on 25 000 simulated and 547 real 512 × 512 grayscale labeled diffraction patterns. The models used five classes for simulated (‘blank’, ‘no-crystal’, ‘weak’, ‘good’, ‘strong’) and two classes for experimental (‘diffraction’ and ‘no diffraction’) diffraction patterns. The training data were made publicly available as the DiffraNet data set (https://dawn.cs.stanford.edu/diffranet/). The classifiers were RF, SVM and a CNN topology based on ResNet-50. The end-to-end CNN could reach 0.985 accuracy on synthetic and 0.945 accuracy on real diffraction patterns. Even though the accuracy of these models is promising for benchmarked data sets, significant hyperparameter tuning and preprocessing are needed. *DeepFreak* uses the BOHB (Falkner *et al.*, 2018 ▸) algorithm for robust hyperparameter optimization, and when optimized for the simulated data set, the network accuracy degraded by at least 22.45% for real images. Local contrast optimization proved essential when using the AlexNet topology, and factors such as background noise and detector artifacts degraded the network performance.

Deep learning has also been used for crystal positioning in X-ray crystallography. Diffraction-based crystal centering induces radiation damage even though the beam is attenuated (Song *et al.*, 2007 ▸). Second-harmonic-generated microscopy has also been used for accurate crystal positioning but requires additional femtosecond IR lasers (Kissick *et al.*, 2013 ▸). The recently developed *DeepCentering* CNN also facilitates cryo-loop and crystal detection in automated centering processes. The system employs a unique object detection algorithm that ignores alterations in the background and also works when the crystal and mother liquor are difficult to distinguish (Ito *et al.*, 2019 ▸). Two programs, *LoopDetector* and *CrystalDetector*, were created as *DeepCentering* components and trained with the Single Shot MultiBox Detector algorithm (Liu *et al.*, 2015 ▸). Although initial training data for *CrystalDetector* were insufficient, the use of various polygon patterns led to effective training. This approach reduced the ambiguity in boundaries between crystals and mother liquor. Crystal detection was considered to be successful when the crystal was within 30 µm of the beam center. The success rate was 90.5% (869/960 images). *DeepCentering* was successful in a fully automated structure determination, including ligand screening. However, *DeepCentering* tended to center on a thinner part of the crystal, compared with manual centering. This resulted in a smaller diffracted volume compared with manual centering, and is a downside of this approach.

Overall, while deep learning offers potential solutions for the improvement of diffraction data collection, there are significant challenges remaining. These include extensive hyperparameter tuning and tailored preprocessing of diffraction patterns. These problems seem tractable, and we consider this aspect of crystallography one of the most promising areas of development. It is likely that, in the not-too-distant future, high-end data collection stations will be controlled by systems that will automatically determine optimal data collection strategies in real time.

### 2.4. Model generation

To determine protein structure one has to infer the phases of the diffraction data, to produce an electron-density map through a Fourier synthesis. Traditionally, phasing techniques such as molecular replacement (Evans & McCoy, 2008 ▸), single isomorphous replacement (SIR) (Blow & Rossmann, 1961 ▸), single-wavelength anomalous dispersion (SAD) (Brodersen *et al.*, 2000 ▸) and multi-wavelength anomalous diffraction (MAD) (Hendrickson, 1991 ▸) have been used. In molecular replacement, the known structure of a homologous protein is used as an initial model to estimate the phases of target protein diffraction data. The emergence of deep learning based protein structure prediction tools like *AlphaFold2* (Jumper *et al.*, 2021 ▸), *RoseTTAFold* (Baek *et al.*, 2021 ▸) and *ESMFold* (Lin *et al.*, 2023 ▸) has opened up new possibilities for phasing. In the 14th edition of CASP (Critical Assessment of Structure Prediction) (CASP14), *AlphaFold2* ranked first on *Z*-score for the Global Distance Test Total Score, which is the average percentage of Cα atoms that are found within certain distance cutoffs of one another between the model and target of four cutoff distances (1, 2, 4 and 8 Å) (McCoy *et al.*, 2022 ▸).

*AlphaFold2* predicts protein structures with remarkable accuracy, significantly advancing the field of protein structure determination. First, it generates a multiple sequence alignment (MSA) of homologous protein sequences by aligning the target protein sequence with its evolutionarily related sequences sourced from publicly available databases. This reveals conserved regions and identifies the sequence variability across different organisms. In the next feature extraction phase, the model generates pairwise residue distances and sequence profiles, replacing commonly used two-dimensional convolution (for details, see Appendix *A*2.3) with an attention mechanism. The extracted features serve as inputs for the deep learning model. These features capture essential information about the protein sequence, such as the conservation of residues, and the co-evolutionary relationships between pairs of residues that might be in close proximity within the three-dimensional structure. The architecture involves an SE(3)-equivariant (Fuchs *et al.*, 2020 ▸) Transformer [an SE(3)-equivariant Transformer is a variant of the self-attention module for three-dimensional point clouds and graphs, and is based on the equivariance with respect to the group of rigid-body transformations in three-dimensional space]; by passing the input through multiple self-attention layers, *AlphaFold2* predicts the three-dimensional structure of the protein. The next iteration updates the pairwise distance and orientation predictions between amino acid residues, refining the predicted three-dimensional structure. *AlphaFold2* provides a measure of confidence for each predicted protein structure, called predicted value of the local distance difference test or pLDDT score. It ranges from 0 to 100, with higher values indicating higher confidence in the predicted structure. The predicted structures can serve as a template for molecular replacement, and can be used to tailor proteins that are difficult to crystallize, by suggesting which parts may be disordered and can be deleted from the protein construct. By using these models, experimentalists can significantly reduce the number of heavy-atom derivatives needed for phasing, streamlining the process and minimizing the cost and time associated with structure determination.

*RoseTTAFold* builds upon the ideas presented by *AlphaFold2*, and is a three-track network integrating information at the one-dimensional sequence level, the two-dimensional distance map level and the three-dimensional coordinate level. The information flows back and forth within these three modalities, progressively transforming and integrating the data, to identify relationships within and between sequences, distances and coordinates. The averaged one-dimensional and two-dimensional features are fed into a final SE(3)-equivariant layer, and end-to-end training directly generates the backbone coordinates. The architecture was developed after CASP14, and was tested on the Continuous Automated Model Evaluation (CAMEO) experiment (Haas *et al.*, 2018 ▸). Out of 69 medium and hard targets, it outperformed all other servers evaluated in the experiment, including *Robetta* (Yang *et al.*, 2020 ▸), *IntFold6-TS* (McGuffin *et al.*, 2019 ▸) and *SWISS-MODEL* (Waterhouse *et al.*, 2018 ▸).

Another recent sequence-to-structure predictor, *ESMFold*, used a Transformer (for details of the Transformer architecture, see Appendix *A*2.5) language model, that resulted in faster and almost equally accurate predictions, compared with *AlphaFold2* and *RoseTTAFold*. A Transformer model called ESM-2, which had up to 15 billion parameters, was trained using a masked language modeling objective. Amino acids were masked out, and the network was trained to retrieve the identities of these masked-out residues, based on the surrounding amino acid sequence. Training was carried out on millions of protein sequences from the UniRef database (Suzek *et al.*, 2015 ▸). By adding an additional module, designed for extracting and structurally interpreting the correlations that ESM-2 had captured, protein structures could effectively be predicted from protein sequences. The *ESMFold* model is a simplification as compared with *AlphaFold2* and *RoseTTAFold* and exhibits significant enhancements in prediction speed, currently at the expense of a slightly lower prediction accuracy.

The integration of *AlphaFold2*, *ESMFold* and *RoseTTAFold* in the phasing process holds great promise for protein crystallography. However, there are still challenges to overcome. For instance, the quality of predicted structures may vary depending on the protein family or the presence of specific domains. Many *AlphaFold2* models have large errors in relative orientations of domains (Read *et al.*, 2023 ▸), although *AlphaFold2* provides warnings in a predicted aligned error (PAE) matrix in these cases. Experimental structures from various crystal forms generally outperform *AlphaFold* models. These models provide a single structure rather than a repertoire of possible conformations that may be influenced by external factors. Nevertheless, the predicted structures are sufficient for molecular replacement in the majority of cases (Millán *et al.*, 2021 ▸).

### 2.5. Map interpretation

Several problems can arise when inferring a protein’s electron density from diffraction data. When phasing by molecular replacement, the resolution of the data may not be high enough for the resulting electron-density map to be unbiased by the model phases, potentially leading to challenges in accurately interpreting the structural details of the target macromolecule. While secondary structures and rigid inner parts of the protein often exhibit clear electron-density maps, side chains and disordered regions are often not visible, which can be attributed to factors like thermal atom vibrations or multiple conformations.

To overcome some of these challenges, Miyaguchi *et al.* (2021 ▸) trained a three-dimensional CNN called *QAEmap* (quality assessment based on an electron-density map) using electron-density maps and their corresponding coordinates as input. They were able to predict the correlation between the local structure and the putative high-resolution experimental electron-density map (Miyaguchi *et al.*, 2021 ▸). They introduced the box correlation coefficient (bCC) as a new metric for evaluating the local quality of protein crystal structures. The method considers the ‘correct’ structure of a protein to be defined by a high-resolution electron-density map, and the bCC serves as a measure of correlation between the coordinates and the electron-density map of the correct structure. The model predicts the bCC even in cases where no high-resolution structure is available. The study compared the performance of the bCC with the real-space correlation coefficient (RSCC). Even though the results suggested that the bCC could offer improvements over existing evaluation methods, it depended on the availability of high-resolution structures as references. bCC potentially neglects broader interactions and structural alignments in the protein, and the presence of multiple conformations and thermal vibrations can lead to a range of potential bCC maxima values.

Human experts and model-building software focus on the distinct shapes of residues for assigning the amino acid sequence of the protein to the electron-density maps. Manually assigning residues can take several days and it still does not ensure that a comprehensive or sufficiently accurate model can be built. As the resolution decreases, it becomes increasingly difficult to observe and differentiate between side chains (Godo *et al.*, 2022 ▸). Popular crystallographic methods are using iterative approaches for model building (Liebschner *et al.*, 2019 ▸; Langer *et al.*, 2008 ▸), while *Buccaneer* uses Bayesian theory for main-chain tracing (Cowtan, 2006 ▸).

3DFC-DenseNet is a three-dimensional CNN architecture capable of directly operating on volumetric data and does not use amino acid sequence information. Data sets of protein density maps at fixed resolutions of 2, 3 and 4 Å were used for training and validation, generating separate models for high-, medium- and low-resolution maps. The evaluation of the model was performed on a data set with varying map resolutions, which were calculated using the experimental X-ray structure factors and phases derived from the atomic model (Godo *et al.*, 2022 ▸). 3DFC-DenseNet could assign amino acid labels to proteins within seconds, outperforming current techniques in the medium-resolution range of 2.5–3.5 Å. It is even effective in the challenging low-resolution range worse than 3.5 Å, where conventional methods struggle. Further addition of residue information into these types of models has the potential to significantly boost the performance.

Building a model in regions of unknown sequence is a challenge. Identifying sequences manually in high-resolution density maps is feasible, but distinguishing between similarly shaped amino acid side chains requires additional information. The task becomes harder at lower resolutions, as model tracing itself is nontrivial without the sequence information (Chojnowski *et al.*, 2019 ▸). To address these issues, researchers developed ‘*findMySequence*’, a ML-based solution, which predicts residue-type probabilities to query sequence databases (Chojnowski *et al.*, 2022 ▸). The network, consisting of two hidden layers, was trained on crystal structures from the Protein Data Bank, where the selection criteria included pairwise sequence identity below 50% and with a resolution between 2 and 3 Å. The model achieved 0.86 accuracy on benchmarked data sets when identifying the most plausible protein sequence.

While existing refinement techniques can introduce restraint conditions from a high-resolution structure of a homologous protein and provide structural checks, subjective decisions are often required and may introduce bias and errors. The described methods enhance processing and interpretation of electron-density maps, and facilitate the determination of protein structures at varying resolutions. They can assist human interpretation of density maps and may provide alternative fittings in poorly ordered density. Structural biology is increasingly being confronted with well phased, but poor-resolution electron-density maps, and we predict that deep learning approaches will have a major impact on the interpretability of such maps.

## 3. Discussion

Overall, the application of deep learning techniques in protein crystallography has shown great promise in improving all stages of protein structure determination. These techniques have the potential to provide automated and efficient approaches to all stages of protein structure determination, which can lead to significant advancements. The models may recommend additives for protein crystallizations. Generative models can be used to predict the optimal conditions for growing protein crystals based on previous experimental data. *AlphaFold2* models have been used to improve protein expression constructs for better crystallization outcomes (https://ccd.rhpc.nki.nl/) (Perrakis & Sixma, 2021 ▸). The implementation of screening in dedicated hardware, such as energy-efficient neuromorphic chips (Esser *et al.*, 2016 ▸), could potentially allow direct integration with data acquisition systems. Although already yielding promising results, deep learning models are still in their early stages of development and face challenges in handling the multi-stage process of protein structure determination through crystallography. Crystallization of proteins is highly dependent on many factors, with high-quality protein purification being a crucial contributing factor. This dependence on multiple variables makes it challenging to achieve consistent outcomes, but deep learning techniques are particularly suited to deal with such high-dimensional data.

Automatic labeling in crystallization outcome prediction and diffraction data collection steps can bypass the inherent variability associated with human labeling. Unlike manual labeling by researchers, computational methods maintain a consistent level of accuracy even across thousands of images, eliminating inconsistencies caused by fatigue. Additionally, inconsistencies arise when multiple crystallographers assign labels to the same data due to variations in their interpretations. However, our review reveals that even with the most advanced nonlinear networks, accurately differentiating between finer-grained classes remains challenging. Expert human reasoning can often prove to be a challenge to model. Recent trends have shifted to using synthetic data sets for supervised learning, which involves creating photorealistic images of virtual protein crystals using ray-tracing algorithms and specialized data augmentations (Bischoff *et al.*, 2022 ▸). The synthetic data sets have been found to result in better-performing models when compared with models fine-tuned with real data, and have also been experimentally validated using high-resolution images from protein crystallization processes. However, in some scenarios, deep learning models fine-tuned to simulated data fail when confronted with real data. Including real data in the training can significantly improve model robustness. Moreover, advancements in imaging capabilities and the introduction of novel systems have the potential to significantly enhance the quality of the data and decision-making abilities of models.

In this context, good-quality data collection, assessment and labeling workflow is not just a preliminary step but the backbone of the entire model development process, playing a pivotal role in ensuring the model’s robustness, reliability and reproducibility. Such a foundation ensures that the model is not overly fitted to a specific subset of the data or overly sensitive to minor variations, thus enhancing its robustness. If achieved through a systematic and efficient workflow, the model performance metrics should be replicable by other researchers using the same data and parameters.

With the advent of deep learning based protein structure modeling, structural biology witnessed a revolution (Read *et al.*, 2023 ▸). Even though *AlphaFold2* knows almost nothing about various factors that can influence protein conformations, these models are already a very useful resource. However, the complexity of Transformer models and their lack of interpretability can impede the identification of crucial features and cannot replace the need for experimental validation of structures and their stoichiometries (Read *et al.*, 2023 ▸).

Deep learning is a relatively young field that is rapidly expanding its applications into many areas. This comes with opportunities, and with risks. We have highlighted the opportunities in our review. Currently, we consider the main risks that cannot be ignored to be underestimation and over-enthusiasm. Underestimation of the potential of deep learning may slow down progress, while over-enthusiasm leads to feeding large data sets indiscriminately into the latest deep learning architecture, with apparently great results that may be very difficult to validate. Also, when prediction models with billions of parameters are being developed and assessed, one might wonder to what extent these can be properly justified. Now that the application of deep learning in the field of protein crystallography is moving beyond the initial exploratory stages, we strongly suggest expanding the scope of the CASP competition beyond comparing structure predictions to also include other fields of structural biology. Good candidates would be crystallization prediction, crystallization trial evaluation, and refinement using experimental data.

## Figures and Tables

**Figure 2:**
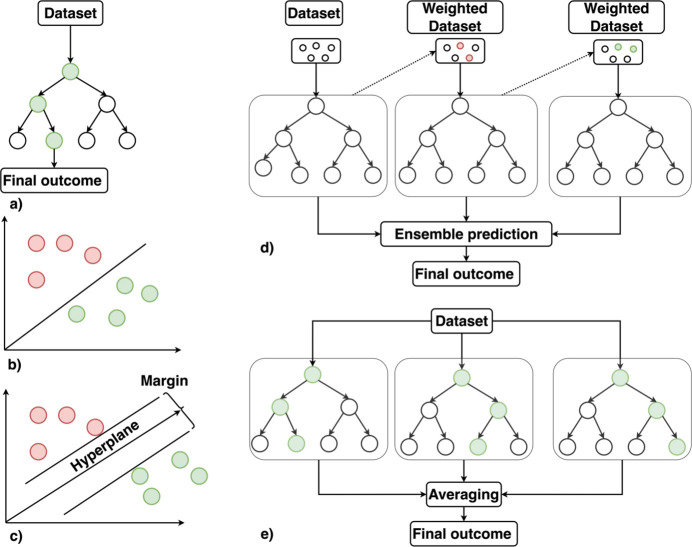
Schematic illustration of (*a*) decision trees, (*b*) naïve Bayes classifiers, (*c*) support vector machines, (*d*) boosting and (*e*) random forest architectures.

**Figure 3:**
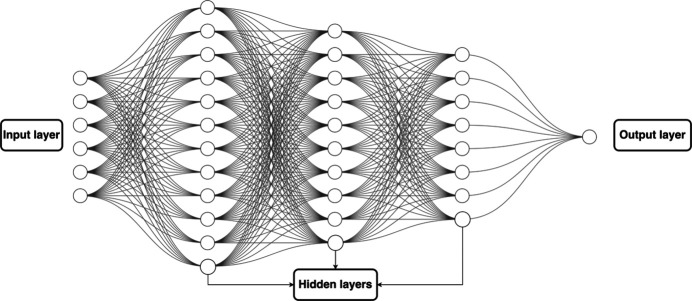
MLP architecture consists of input, output and hidden layers.

**Figure 4:**
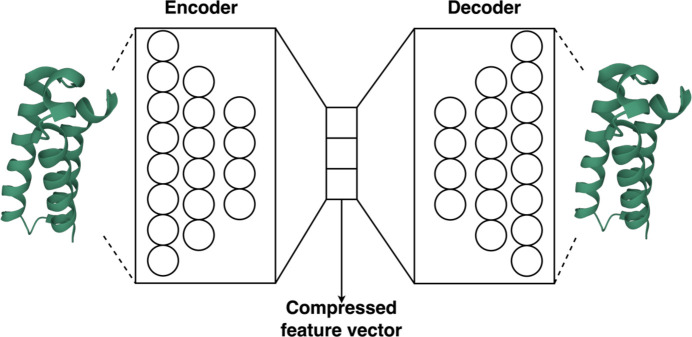
The encoder and decoder parts of the autoencoder network. The encoder transforms the input features into low-dimensional space, which then can be retrieved by the decoder network.

**Figure 5:**
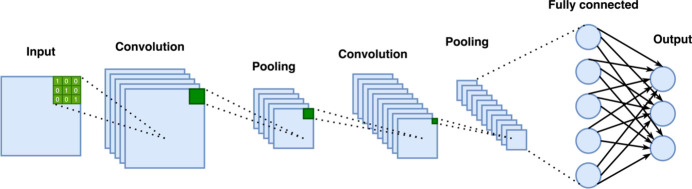
Schematic illustration of a CNN architecture that generates probabilities, indicating how likely it is that input data belong to a specific class. The input image is convolved with a kernel (green), and the resulting feature map is propagated through the network.

**Figure 6:**
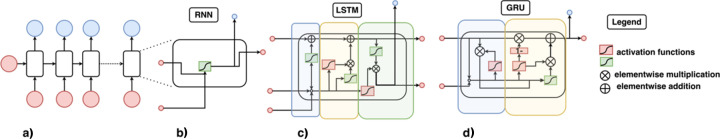
The main building blocks of RNNs, LSTMs and GRUs. (*a*), (*b*) The main building block of RNN. The previous step information is added to the current input and passed through the activation function. The output is then used for the prediction and passed to the next timestep. (*c*) LSTM uses gating mechanisms (for details, see Appendix *C*4) to control the flow of information through the hidden state and has three gates. The forget gate controls how much information from the previous timestamp should be forgotten. The input gate controls how much of the new input should be added. The third gate passes the current cell state to the next timestep. (*d*) The GRU uses a similar mechanism to the LSTMs; however there are only two gates.

**Figure 7:**
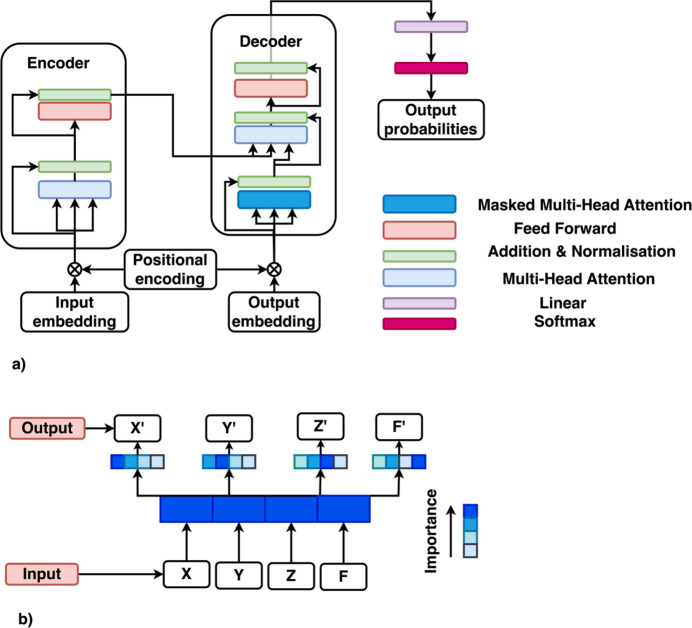
(*a*) The encoder and decoder blocks of the Transformer. The architecture employs a self-attention mechanism to efficiently process sequences in parallel. Multi-head attention and positional encodings provide the model with rich contextual information. (*b*) The attention mechanism. The sequence of *X*′, *Y*′, *Z*′ and *F*′ is generated based on the importance or attention to specific input tokens *X*, *Y*, *Z* and *F*. The attention weights are calculated using a compatibility function that measures the relevance of each input to the output being generated.

**Figure 8:**
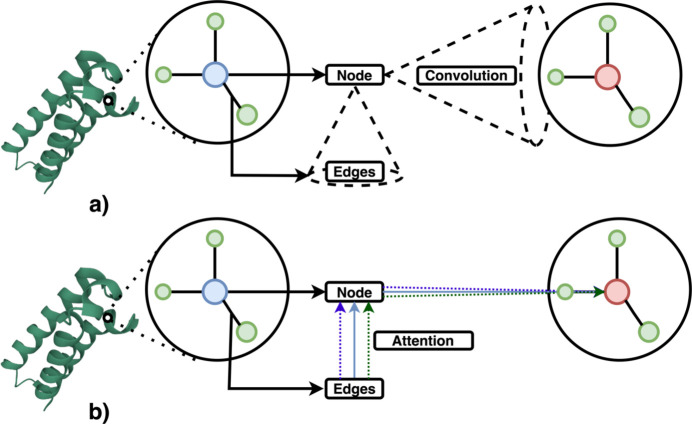
Schematic illustration of convolution operation and attention mechanism on protein graphs. (*a*) In GCNNs convolution is described as having a receptive field of neighboring residues and the activation updates the center residue. (*b*) In GATs, the influence of each interaction can be adjusted in order to capture more nuanced and context-dependent interactions.

**Figure 9:**
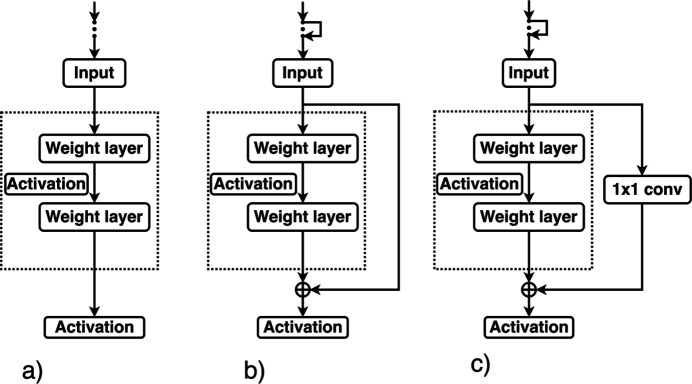
The classical (*a*) and residual (*b*), (*c*) mapping of the input. (*a*) In a classical CNN, the network directly learns input to the output mapping function, passing the result to the activation function. (*b*), (*c*) In ResNets the network primarily learns the residual or difference between the input and the output, as opposed to the complete output mapping, either by identity mapping or by a so-called 1 × 1 convolution that collapses one or more dimensions. This approach enables each layer to capture additional nuances without losing the information learned by previous layers.

**Figure 10:**
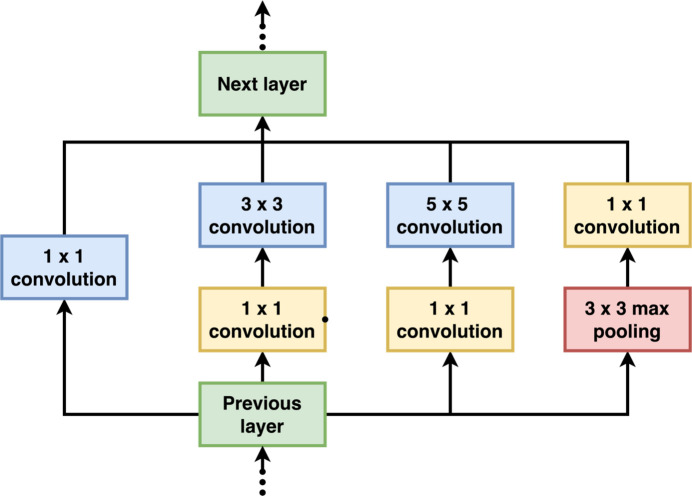
Inception module with dimension reductions described in GoogLeNet. Parallel branches perform different types of convolutions with varying kernel sizes and pooling operations.

## Footnotes

1 Data augmentation refers to the technique of artificially expanding the size and diversity of a training data set. Various transformations are applied to the existing data samples, generating altered versions of them. Common augmentations for image data include rotations, scaling, flips, cropping and color adjustments.

## Appendix A ML architectures

### A1. Traditional ML techniques

*Naïve Bayes* is a class of probabilistic classifiers based on Bayes’ theorem with the strong assumption that each input variable is independent of the other variables. Despite this simplification, the model is easy to build, particularly for large data sets, and it is fast compared with more sophisticated methods for classification tasks (Fig. 2 ▸).

*Decision trees* recursively split the input data into smaller subsets based on a set of rules until a stopping criterion is reached. Such decision trees are used for both classification and regression tasks. Random forests (RF) are an extension of decision trees that combine multiple decision trees to improve their performance (Song & Lu, 2015 ▸). By constructing multiple trees on randomly sampled subsets of the input data and aggregating their predictions, random forests aim to improve accuracy. However, decision trees and random forests are prone to overfitting, especially if the tree architecture becomes complex.

*Boosting* is a technique that improves the performance of weak classifiers by iteratively training weak classifiers on different subsets of the input data and assigning a higher weight to misclassified samples in each iteration (Friedman, 2001 ▸). The goal is to combine these weak classifiers into a strong classifier. Boosting is sensitive to the choice of weak classifiers and the number of iterations, and careful consideration must be given to these factors to achieve optimal results. Gradient boosting, being the advanced form of the boosting technique, identifies the shortcomings of weak classifiers by using gradients in the loss function. It leverages the concept of gradient descent (for details, see Appendix *C*5), and at each iteration, a new weak classifier is trained not just on the misclassified samples, but on the residuals or errors of the previous prediction.

*Support vector machines* (SVMs) aim to find the hyperplane that maximizes the margin between the different classes in the input data (Cortes *et al.*, 1995 ▸). (Hyperplanes are decision boundaries for the classification of data points. Data points falling on either side of the hyperplane can be attributed to different classes. The dimension of the hyperplane depends on the number of features in the input data set.) The margin is defined as the distance between the hyperplane and the closest data points from each class. SVMs can also transform the input data into a higher-dimensional space, where it may be easier to separate the different classes. However, this transformation can lead to nonlinear behavior. SVMs can handle high-dimensional data sets but can be computationally expensive.

### A2. Deep learning architectures

#### A2.1. Multi-layer perceptrons

Multi-layer perceptrons (MLPs) are supervised learning algorithms, and are known as the foundation architecture of deep learning. A typical MLP consists of an input layer that receives input data, and an output layer that makes a prediction (Fig. 3 ▸). The processing in hidden layers is nonlinear, with each node generating an output value based on its input values and some internal parameters that are optimized by training with characterized data and the ground truth (Karhunen *et al.*, 2015 ▸). Training involves passing data through the network with the nodes having initial, suboptimal parameters. This forward pass results in predictions for a given input. Especially early in the training, these predictions have errors reflecting the differences between the predictions and the ground truth. By reversing the errors back into the network through backpropagation, the gradient of the loss function (for details, see Appendices *C*2 and *C*3) with respect to the parameters of the network is calculated. This allows optimization of the network’s parameters by steepest descent or conjugate gradient methods. This iterative process is repeated until the model converges to a minimum of the loss function. The number of layers and nodes varies based on the specific task. MLPs having enough nodes in a single hidden layer can approximate any smooth enough nonlinear input–output mapping.

#### A2.2. Autoencoders

Autoencoders (AEs) are deep latent models consisting of two subnetworks: encoder and decoder (Fig. 4 ▸). (The term ‘latent space’ refers to hidden or underlying features or variables in data which are not directly observable, but can be inferred or learned through ML models.) The encoder network learns to map data points to low-dimensional, compressed feature vectors, while the decoder network learns to reconstruct data points from their low-dimensional latent-space representation (Hawkins-Hooker *et al.*, 2021 ▸). In protein crystallography AEs are mainly used to learn the representation of the content of protein sequences or transform huge numbers of protein-specific input parameters into corresponding, compressed features.

#### A2.3. Convolutional neural networks

A convolutional neural network (CNN) is a supervised deep learning neural network initially designed for image and video recognition tasks (Cun *et al.*, 1990 ▸). It consists of stacked convolutional and pooling layers, which work together to learn and extract hierarchical features from input data in an adaptive and automatic way (Fig. 5 ▸).

In a CNN, a convolutional layer applies a set of learnable filters or kernels to the data. In the case of image data, a kernel is a small matrix, which slides across the image and computes a dot product between its weights and the corresponding local field of the input data, resulting in a feature map. Thus, it maps the location and strength of specific features – like edges, textures or shapes in the input data. A pooling layer down-samples its input data while retaining the most salient features. This is done by dividing the input into non-overlapping regions and computing a summary statistic, such as the maximum or average, for each region. The output of a pooling layer typically has smaller dimensions than the input (Fig. 5 ▸).

Once the data have passed through the last pooling step, they are flattened into a one-dimensional array. This array is multiplied by a weight matrix in the fully connected layer and passed through an activation function, which produces a probability score for each specific class. The probabilities that a CNN assigns to its set of potential classifications depend on the kernel weights, pooling parameters and weight matrices.

#### A2.4. Recurrent neural networks

Recurrent neural networks (RNNs) process sequential data (Lipton *et al.*, 2015 ▸), such as time series or natural language processing (NLP) tasks. Unlike other types of neural networks, RNN nodes each have a ‘memory’ in the form of a hidden state, which allows them to maintain and propagate information about the previous inputs in a sequence (Fig. 6 ▸). The output of a node in the network therefore depends not only on the current input but also on previous inputs. When RNNs have many layers, training can become problematic when the gradients of successive layers must be multiplied. When successive weights are all low (or high), the gradients vanish (or explode) (for details, see Appendix *C*6), resulting in instabilities and failure to converge. To minimize such problems, long short-term memory (LSTM) and gated recurrent units (GRUs) were introduced.

In LSTMs and GRUs, the weight of previous inputs is controlled by the gating mechanisms, allowing the models to selectively remember or forget information from previous inputs (Fig. 6 ▸). The contributions of recent and earlier events are determined by the gating mechanisms, rather than a progressive reduction in the weight of previous inputs. This allows the network to ‘remember’ significant long-range patterns, and ‘forget’ insignificant ones (Fig. 6 ▸). GRU nodes simplify the LSTM structure by combining the forget and input gates into a single gate.

LSTMs can therefore be more effective at capturing long-term dependencies in sequential data than GRUs, but they tend to be more computationally expensive.

#### A2.5. Transformers

The Transformer architecture was first introduced by Vaswani *et al.* (2017 ▸). It revolutionized the field of NLP and found applications in various other domains including protein structure prediction. Basic data structures, referred to as ‘tokens’, play a crucial role in NLP tasks as they provide the basis for the model to learn patterns and relationships. In the context of language, tokens can be words, while in protein structure prediction, tokens can be amino acids.

The Transformer relies on the self-attention mechanism (for details, see Appendix *A*2.7), which captures dependencies between tokens without relying on recurrent or convolutional layers, making it possible to process entire sequences in parallel. The encoder and decoder in the Transformer architecture comprise multiple identical layers (Fig. 7 ▸). The encoder processes input sequences, and is connected to the decoder through an attention mechanism, to facilitate information flow between them. In Transformers, due to the introduction of the multi-head attention mechanism, the network attends to different parts of the input sequence simultaneously, capturing various aspects of the context. As in this scenario the order of tokens in the input sequence is not preserved, the Transformer uses positional encoding, which helps the model to infer the position of each token in the sequence.

#### A2.6. Attention mechanism

The attention mechanism is a technique used in deep learning models to selectively focus on specific aspects of the input data (Vaswani *et al.*, 2017 ▸). It was initially introduced to improve the performance of RNNs in capturing long-range dependencies between input and output data.

The attention mechanism computes a set of weights or correlations between the input data and the output data, using the hidden states of the RNN nodes. These weights allow the model to focus on the most relevant parts of the input sequence, regardless of their spatial distance, effectively enabling the model to capture long-range dependencies [Fig. 7 ▸(*b*)].

By giving more weight to the specific features or patterns of the training data that correlate well with the desired output, the attention mechanism allows the model to selectively emphasize the most informative aspects of the input data, thereby improving its performance in a variety of tasks. The attention mechanism is widely adopted in sequence-based models, such as Transformers and graph attention neural networks, among others.

#### A2.7. Self-attention mechanism

A self-attention is an attention mechanism that computes attention scores for all parts of the input relative to all other parts. This means that every part of the input has the potential to influence every other part in the output, which makes self-attention particularly powerful for handling complex dependencies within the data. Furthermore, multiple ‘heads’ of the self-attention mechanism can operate in parallel. In this scenario, each head can perform parallel computing, which means that the computation time will be greatly reduced.

#### A2.8. Graph convolutional neural networks

Graph convolutional neural networks (GCNNs) generalize the convolution operation from regular grids, such as images, to graph-structured data. In a GCNN, the convolution operation is replaced with a graph convolution operation that takes into account the topology of the graph, as well as the features of the nodes and their neighbors. In GCNNs, a protein may be represented as a graph, where the nodes may contain the amino acid residue information of the protein with various embedding features assigned to it, and the edges are the interactions between the residues [Fig. 8 ▸(*a*)].

#### A2.9. Graph attention neural networks

While GCNNs leverage graph topology and node features for network learning, graph attention neural networks (GATs) leverage the attention mechanism that allows nodes to weigh the importance of their neighbors. In GATs, each node in the network can focus on different neighboring nodes and capture more nuanced and context-dependent interactions. In this case, when representing the protein, the influence of each interaction (edge) between residues can be adjusted based on the context, allowing a more flexible and adaptive model [Fig. 8 ▸(*b*)].

#### A2.10. Residual neural networks

Residual neural networks (ResNets) were developed to solve the vanishing gradient problem (for details, see Appendix *C*6) in deep CNNs for image analysis (He *et al.*, 2016 ▸). They allow efficient training of very deep networks (*e.g.* hundreds of layers) without sacrificing network performance. They work by adding the weighted input of a layer to the output of a subsequent layer. This can greatly facilitate training, as it allows non-discriminating nodes to be skipped when calculating the gradients through backpropagation (Fig. 9 ▸).

#### A2.11. Inception convolutional neural networks

Inception networks use several convolutional kernels with different sizes in the same layer, unlike traditional CNNs (Szegedy *et al.*, 2016 ▸). This design enables the network to adaptively learn features at different scales and complexities. Their core component is the inception module, consisting of parallel branches that perform different types of convolutions with varying filter sizes (*e.g.* 3 × 3, 5 × 5) and pooling operations (Fig. 10 ▸). The outputs from these branches are concatenated which increases dimensionality. Dimensionality is reduced in 1 × 1 convolutional layers, which are projections that compress the feature maps while preserving spatial information. Auxiliary classifiers address the vanishing gradient problem that can occur in deep architectures. These classifiers provide additional supervision during training, encouraging the network to learn more discriminative features. Inception networks are used for image classification, object detection and semantic segmentation. Since the introduction of the Inception architecture, several variants and improvements have been proposed, such as Inception-V2, Inception-V3 and Inception-ResNet (Szegedy *et al.*, 2016 ▸). These updated versions incorporate advanced techniques like batch normalization, factorized convolutions and residual connections to further enhance the performance and efficiency of the network.

## Appendix B Evaluation metrics

### b1. Classification metrics

*Accuracy* is used to evaluate the performance of a classification model. It is defined as the ratio of the number of correct predictions made by the model to the total number of predictions. It can be expressed as

*Precision* is defined as the proportion of true positive predictions among the positive predictions made by the model. Mathematically, it is expressed as

*Recall* is a measure of the quantity of the positive predictions. It is defined as the proportion of positive instances that were correctly identified by the model and is expressed as

*F1-Score* provides a balance between precision and recall. It is defined as the harmonic mean of precision and recall:

*AUC-ROC* (area under the receiver operating characteristic curve) is a widely used metric for evaluating the performance of a binary classification model. AUC-ROC is defined as the area under the receiver operating characteristic (ROC) curve, which plots the true positive rate (TPR) against the false positive rate (FPR) at various classification thresholds. AUC-ROC ranges between 0 and 1, where a value of 1 represents a perfect classifier and a value of 0.5 represents a random classifier.

*Matthew’s correlation coefficient* (MCC) takes into account true positives, false positives and true negatives and is generally regarded as a balanced measure since it can be used even if the classes are of very different sizes. The MCC returns a value between −1 and +1. A coefficient of +1 represents a perfect prediction, 0 no better than random prediction, and −1 indicates total disagreement between prediction and observation. The MCC is calculated using the formula

where TP is the number of true positives, TN is the number of true negatives, FP is the number of false positives and FN is the number of false negatives.

### b2. Regression metrics

The *mean absolute error* (MAE) measures the average magnitude of the errors in a set of predictions, without considering their direction:

The *mean-squared error* (MSE) measures the average squared difference between the predicted and actual values. RMSE is the square root of the mean-squared error and is expressed in the same units as the target variable,

The *R-squared (coefficient of determination)* measures the proportion of variance in the target variable that is explained by the model. Mathematically, it can be expressed as

where *n* is the number of observations, *y_i_* is the target value,  is the predicted value for the *i*th observation and  is the average of the observed data.

## Appendix C Model details

### c1. Activation functions

Activation functions introduce nonlinearities into the network’s computations. This allows the network to learn from complex data. An activation function processes the weighted sum of the inputs, alongside a bias, then determines whether a neuron should be activated based on the input.

*Sigmoid function*. The sigmoid function, represented as  , outputs a value between 0 and 1. In a binary classification neural network, the output of the sigmoid function can be interpreted as a probability for binary class.

*Hyperbolic tangent or tanh function*. The tanh function, represented as  =   , is similar to the sigmoid function. However, the outputs are in a range between −1 and 1. This can be advantageous because the negative inputs will be mapped strongly negative, and the zero inputs will be mapped near zero.

*Rectified linear unit or ReLU*. The ReLU function, represented as  , is one of the most commonly used activation functions in deep learning models. The function returns 0 if the input is negative, and the value for any positive input is *x*.

Each of these functions can be utilized in different scenarios based on the specific requirements and the neural network architecture.

### c2. Loss function

A loss function (or cost function) *L*, in the context of neural networks, is a function that calculates the difference between the predicted output  and the true output *y*. The goal of training a neural network is to minimize the loss function. As an example, in regression tasks, a common loss function is the mean-squared error (MSE; see Appendix *B*2), which is calculated as

where *N* is the number of observations,  is the true output for the *i*th observation, and  is the predicted output for the *i*th observation.

### c3. Backpropagation

Backpropagation is an algorithm to calculate the gradient of the loss function with respect to the weights in the neural network. This is done by backpropagating the error of the output layer through the network layers, starting from the output layer and moving back towards the input layer. If we have a loss function *L* and weights *W*, we calculate  for this purpose. The weights of the network are then iteratively updated in the negative direction of the gradient to minimize the loss.

### c4. Gating mechanism

In LSTMs and GRUs a gating mechanism is used to control the flow of information. A gate in these networks is usually a sigmoid layer and a pointwise multiplication operation. Mathematically, a gate *g* can be expressed as *g* =  ; here σ is the sigmoid activation function, *W* and *U* are the weight matrices, *x* is the input, *y* is the previous output and *b* is the bias term. The output of the sigmoid function, which ranges from 0 to 1, determines whether a particular piece of information is allowed to pass through (close to 1) or blocked (close to 0).

### c5. Gradient ascent and descent

Gradient ascent and descent are utilized to find local minima and maxima of a function iteratively. The calculation of the gradient or derivative of the function at a specific point gives the direction of steepest ascent or descent.

The parameters of the function are adjusted in the opposite direction of the gradient to minimize the output in the case of gradient descent and in the same direction in the case of gradient ascent. These adjustment steps are defined by a parameter called the learning rate, in order not to overshoot the optimum point.

### c6. Vanishing and exploding gradients

The vanishing gradients problem arises when the gradients of the loss function become very small with respect to the weights of the network. In deep layers of the network the gradients of the loss function can decrease exponentially with the depth of the network during backpropagation. Consequently, the weights in the earlier layers of the network are updated very little during each iteration of gradient descent, slowing down learning or causing it to stop entirely. This problem is even more pronounced when activation functions squash their inputs into a narrow range.

Conversely, the exploding gradients problem refers to the situation when the gradients become too large, causing the updates to the weights during gradient descent to be extremely high. In this scenario the network becomes unstable and fails to converge.

Several techniques, like gradient clipping, weight initialization strategies, batch normalization and the use of different activation functions, have been developed to mitigate these problems.
